# Ideal Point Modeling of Non-cognitive Constructs: Review and Recommendations for Research

**DOI:** 10.3389/fpsyg.2018.02423

**Published:** 2018-12-10

**Authors:** Louis Tay, Vincent Ng

**Affiliations:** Department of Psychological Sciences, Purdue University, West Lafayette, IN, United States

**Keywords:** ideal point, personality measurement models, non-cognitive, test construction, item response process

## Abstract

Most psychological measurement models assume a monotonically increasing relation between the latent trait and the probability of responding. These models have historically been based on the measurement of abilities (e.g., cognitive), which have dominance properties. However, they are less appropriate for the measurement of non-cognitive constructs, or self-reported typical behaviors (e.g., attitudes, emotions, interests, personality), which have historically been assumed to have ideal point properties, or a nonmonotonic relation between the latent trait and the probability of responding. In this paper, we review the literature on ideal point modeling of non-cognitive constructs to present a theoretical framework that can help guide researchers on pertinent factors that may influence ideal point responding when assessing non-cognitive constructs. We also review the practical implications of not using ideal point response models for non-cognitive constructs and propose areas for research in non-cognitive construct assessment.

## Introduction

The field of psychometrics has historically distinguished between the item response processes underlying the assessment of non-cognitive constructs and cognitive constructs (Spearman, 1904; Thurstone, 1928). Early on, this led to the development of two different types of measurement models. We will discuss the core differences in later sections; for now, it is sufficient to note that cognitive constructs were assessed with *dominance models* such as factor analyses and logistic item response theory (IRT) models. On the other hand, non-cognitive constructs were assessed with *ideal point models*. However, to date, the measurement models used for assessing non-cognitive constructs—including attitudes, affect, personality, and interests—have been based on dominance models originally developed for cognitive constructs. In this paper, we trace the reasons for this and show how this historical distinction still holds relevance for psychology. We present theoretical and practical reasons for the continued development of ideal point measurement models and discuss future areas for research.

### Non-cognitive Assessments: Past to Present Application of Ideal Point Models

Historically, cognitive and non-cognitive assessments were developed with a recognition that there were marked distinctions in how individuals made responses to assessment items (Tay et al., 2009; Drasgow et al., 2010). Dominance models were developed for assessing cognitive ability constructs because it was assumed that higher cognitive ability level would lead to a monotonically increasing probability of a higher score. Indeed, the term “dominance” was coined by Coombs (1964) to express how individuals with higher trait levels invariably dominate or overcome assessment items by correctly answering them. The presumption of a dominance model is reflected in the use of factor analysis for cognitive ability (Spearman, 1904), which conforms to the dominance assumption. Within the field of psychometric testing, other types of dominance models stemming from Thurstone’s descriptive plots of response curves for the Binet-Simon test (Thurstone, 1925) were also developed within the IRT framework, including cumulative normal ogive and logistic IRT models (Bock, 1997).

Ideal point measurement models, or ideal point models, were initially developed for non-cognitive constructs (Coombs, 1964). Unlike dominance models, the response curve in ideal point models is assumed to be nonmonotonic with respect to the latent trait. This can be seen in the assessment of attitudes: Thurstone (1928) proposed the use of an ideal point model within which a higher latent level on the trait was not necessarily monotonically related to the probability of a higher observed score. Instead, the probability of respondents selecting the highest observed score option for a given item would be a function of their latent trait level *matching* the item level (Roberts et al., 2000). Individuals whose latent trait level is either too far above or below the item location would be less likely to express strong endorsement. A substantive example would be political attitudes where individuals tend to endorse (or agree most strongly with) items that reflect their political views. Having a higher latent level would not necessarily lead to a higher observed score on a political statement. Instead, a statement is endorsed to the extent that it is closest to the latent trait level so that political moderates would tend to most strongly endorse more moderate political statements, but less strongly endorse statements reflecting more extreme positions along the latent continuum.

Despite this seminal distinction, the use of Likert-type scaling and reverse scoring has obviated the use of more complicated ideal point scoring procedures in which items all had to be scaled based on participant ratings prior to scoring (Thurstone, 1925, 1927a,b). Through the use of Likert-type scaling (e.g., 1 – “strongly disagree” to 5 – “strongly agree”), reverse-scoring scale items on the opposite end of the construct continuum (i.e., reverse-worded items), and excluding middling items on the continuum, one can more simply summate the ratings to produce a reliable score that distinguishes individuals on attitudes—and other non-cognitive constructs (Likert, 1932). This convenience in scoring led to the decline of the ideal point model and the greater use of dominance models for assessing attitudes (Brayfield and Rothe, 1951; Schreiber et al., 1952) and also other non-cognitive constructs such as personality (Lovell, 1945; Tyler, 1951). To the present day, most of the measurement models applied to non-cognitive constructs rest on dominance response assumptions.

Nevertheless, ideal point type measurement models have been developed over time due to historical interest (Zinnes and Griggs, 1974; Andrich and Luo, 1993). The most recent generalizable model that allows for the parameterization of the width and peak of the ideal point IRT curves is the Generalized Graded Unfolding Model (GGUM) by Roberts et al. (2000). The development of accompanying statistical approaches and software for estimating the GGUM and scoring individuals has also lowered the barriers for applying these models to non-cognitive constructs (Roberts, 2001, 2008; Roberts et al., 2002; Roberts et al., 2004). These advancements now enable researchers to apply these models to a variety of non-cognitive constructs. But are there theoretical and substantive benefits of applying ideal point models to non-cognitive constructs? Are there downsides to applying dominance models to non-cognitive constructs?

### Consequences of Applying Dominance Models to Non-cognitive Constructs

At present, the extant literature suggests that applying dominance models to non-cognitive constructs leads to mixed evidence for effects and confusion in two areas: dimensionality of bipolar constructs and curvilinear relationships between positive constructs and positive outcomes. First, ideal point modeling makes a critical difference in uncovering the unidimensionality of bipolar, non-cognitive constructs. It has been mathematically shown that the inappropriate application of dominance modeling (i.e., principal components analysis) to ideal point response data leads to the recovery of two components instead of one component, the second of which is a spurious factor reflecting the item response process (Davison, 1977). This may not be too surprising if one considers that individuals whose latent trait level is in the middle of the continuum (e.g., moderates on a political attitude scale) would tend to co-endorse items located close to and on either side of the middle of the continuum, leading to a smaller-than-perfect (negative) correlation between the two ends of the continuum. This may be mistakenly modeled as two components or factors rather than a single bipolar continuum using a dominance approach.

How might incorrectly modeling bipolar non-cognitive constructs with dominance methods affect substantive research? As identified by Greenwald (2012), one of the longest standing controversies in psychology for over 50 years is the bipolarity of affect—whether happiness and sadness are two orthogonal factors or if they are part of a single bipolar factor (Bradburn, 1969). Based primarily on factor analytic evidence, many researchers have held to the idea that happiness and sadness are orthogonal (Watson and Tellegen, 1985; Cacioppo and Bernston, 1994; Larsen et al., 2009). However, others hold to the theoretical idea that happiness and sadness fall on a bipolar continuum (Russell, 1979). Recent research on this issue suggests that individuals use an ideal response process when responding to emotion indicators (Tay and Drasgow, 2012). Moreover, because individuals tend to be in the middle of the bipolar continuum, they more strongly endorse indicators in the middle of the continuum (e.g., both “slightly unhappy” and “slightly happy”). This leads to attenuation of the correlation between happy and sad and also results in non-negligible co-endorsements of happiness and sadness (Tay and Kuykendall, 2016).

Second, there is an emerging interest within psychology in detecting curvilinear effects (Carter et al., 2017), especially between theoretically positive predictors and outcomes (Grant and Schwartz, 2011; Pierce and Aguinis, 2011). Yet, the support for curvilinear effects has been mixed. For example, Carter et al. (2014) found in a review of the literature that just under half of the studies investigating the curvilinear (i.e., inverted U-shape) relationship between conscientiousness and job performance found empirical support for the effect, but all of these studies used a dominance model scoring (e.g., sum scores). They further showed using data collected from consulting firms that ideal point model scoring revealed curvilinear relationships between conscientiousness and various job performance measures more often (100%) than dominance model scoring (37.5 and 75%).

Carter et al. (2016) similarly found that curvilinear relationships between conscientiousness and well-being measures (i.e., life satisfaction, positive and negative affect, job satisfaction, and work stress) were detected more often using ideal point model scoring compared to dominance model scoring. These results indicate that one reason why there is such mixed evidence for curvilinear effects is due to the misapplication of dominance models when scoring non-cognitive constructs that respondents may be responding to using an ideal point process. One should note however that research using ideal point and dominance models to assess curvilinear relations has also at times not found practical differences (Wiese et al., 2018).

Simulation work has also found that when data followed an ideal point model, the application of ideal point models demonstrated higher power and greater accuracy in estimates of curvilinear effects as compared to dominance approaches (Cao et al., 2018). It is also important to note that ideal point models can also be misapplied—to data where respondents are responding using a dominance process. Carter et al. (2017) found in simulation studies that when ideal point scoring was applied to simulated data reflecting a dominance response process that errors in detecting curvilinearity occurred. Specifically, they found high Type 2 errors: under conditions where there was no curvilinear relationship, ideal point scoring led to the positive values for the curvilinear effect coming back as statistically significant under two-tailed tests. More recent work by Cao et al. (2018) suggests that ideal point models may still be able to be as powerful and accurate as dominance models when applied to larger sample sizes of around 2,000 individuals. This is likely due to the more general form of the ideal point model as compared to dominance models.

The emerging benefits of ideal point measurement models for non-cognitive constructs has led to a resurgence of interest on this topic, but also new questions. What are the theoretical foundations of an ideal point response process for non-cognitive constructs? Based on this theoretical framework, when can we expect ideal point response processes to most likely occur? Are there practical consequences of misrepresenting the ideal point response process? Addressing these questions can provide a roadmap for psychometric research in non-cognitive constructs.

### Theoretical Framework Underlying Ideal Point Response Models

Issues regarding validity have been significant in the field of psychometrics (Cronbach and Meehl, 1955). This is because the assumptions made about the theoretical constructs we seek to measure can fundamentally affect the validity of the types of measurement models used and how such scores are interpreted (Borsboom et al., 2003, 2004). Consequently, it is necessary to unpack the theoretical differences between cognitive and non-cognitive constructs and their assessment. In discussing these differences, we do not intend to make philosophical claims about the fundamental nature of these constructs *per se* (Borsboom et al., 2003). In a similar fashion to formative and reflective constructs, which seek to understand the relationship between indicators and constructs (Edwards and Bagozzi, 2000), we seek to unpack why and how the relationship between indicators and constructs is better understood either in a dominance (i.e., “S” shaped response curve) or ideal point response (i.e., inverted-“U” response curve) fashion. We draw on past theoretical work to develop an integrative framework (Tay et al., 2009; Tay and Drasgow, 2012). This framework is summarized in Table 1, which we hope can provide a roadmap for these issues.

**Table 1:** Key distinctions between cognitive and non-cognitive constructs.

	Cognitive	Non-cognitive	Areas for future research
Characteristic definition	Maximal performance behaviors	Self-reported typical behaviors	What types of non-cognitive constructs display the strongest ideal point response processes?
Goal of assessment	To capture psychological threshold or limits	To capture habitual characteristics, typical modes of action, or descriptive states	Do individuals perceive these assessment goals and how do they relate to ideal point responding?
Ideal output of high assessment motivation	Maximum capacity	Maximum accuracy	What types of assessment situations (e.g., high stakes vs. low stakes; high motivation vs. low motivation) lead to more ideal point response processing?
Meaning of construct continuum	Difficulty	Location	—
Polarity	Unipolar	Unipolar or bipolar	To what extent does the polarity of construct impact the response process used?
Item response process and model	Hurdle algorithm Dominance models	Matching algorithm Ideal point models	Are there intraindividual ways to assess the use of a hurdle or matching algorithm?

#### Characteristic Definitions

Before we discuss how response processes differ between cognitive and non-cognitive constructs, it is important to provide greater clarity on the classes of constructs for which dominance and ideal point response processes are most likely to occur. While cognitive and non-cognitive constructs are used as first approximations in nomenclature, the labels of “maximal performance behaviors” and “self-reported typical behaviors,” respectively, more precisely capture the distinctive essence of these types of constructs (Tay et al., 2009; Tay and Drasgow, 2012). The approach to assessing maximal performance behaviors is one that presents challenges to individuals in the form of test items to ascertain their maximum ability. For cognitive constructs, the assessments are meant to challenge individuals to their psychological thresholds or limit. This is a common occurrence in cognitive ability tests. By contrast, the approach of assessing self-reported typical behaviors is one that presents opportunities to individuals to agree or disagree to descriptive statements as is frequently encountered in non-cognitive construct measures. The goal of this type of assessment is to capture habitual characteristics, typical modes of actions, or descriptive states.

One reason for using the terms maximal performance and self-reported typical behaviors as characteristic definitions is that cognitive and non-cognitive constructs can be construed and assessed in formats/situations that may not produce distinct dominance or ideal point response processes. Dimensions of cognitive ability can be, and has been, assessed in a non-cognitive self-report manner (e.g., self-report spatial ability; Hegarty et al., 2002). Similarly, non-cognitive constructs can be assessed in a manner that is analogous to performance (e.g., moral ability; Rest, 1986). Another reason is that there are cognitive and non-cognitive constructs that fall out of the purview of the dominance and ideal point response models, such as constructs that are either implicit by definition or best assessed using indirect methods and thus less explicitly processed during assessment (Greenwald et al., 1998; James and LeBreton, 2012). This characteristic definition can help researchers understand how only some types of cognitive or non-cognitive constructs (and related assessment procedures) are most likely to produce different types of response processes. For the rest of the paper, while the terms maximal performance and self-reported typical behavior constructs are more precise, for the sake of simplicity and consistency, we will generally refer to them as cognitive and non-cognitive constructs, respectively.

#### Ideal Output of High Participant Motivation

The difference in characteristic definitions of and goals of assessment for cognitive and non-cognitive constructs (capturing psychological limits versus tendencies, respectively) leads to corresponding differences in the ideal output of assessment. At the outset, the definitions of maximal performance behaviors and self-reported typical behaviors used above to characterize cognitive and non-cognitive constructs should not be confused with maximal performance and typical performance (Sackett et al., 1988). In speaking of maximal performance and typical performance, we are discussing different motivational *levels*, which produce peak performance as compared to daily performance. However, with maximal performance behaviors and self-reported typical behaviors, we are assuming here similarly high motivational levels during the assessments that produce distinct motivational outputs. For cognitive constructs, high motivation produces *maximum capacity* to overcome test items, whereas for non-cognitive constructs high motivation produces *maximum accuracy* to agree or disagree to self-descriptive statements.

We recognize that high participant motivation may not necessarily lead to maximum capacity or maximum accuracy for cognitive and non-cognitive constructs, respectively; these represent the ideal outputs to produce the expected response processes. For non-cognitive constructs, which are of primary interest, there are testing situations in which high motivation may potentially lower accuracy. For example, in high-stakes testing situations, individuals may view assessment items as performance-based assessments, which could reduce an ideal point response process and shift response processes to be more dominance-like (cf. O’Brien and LaHuis, 2011). Perhaps a distinction should be made between high motivations to follow assessment directions as opposed to engage in impression management. Indeed, it is likely that in situations in which self-presentation bias or faking occurs, the response processes may change. Another implication is that in assessment situations where there is low motivation to complete a non-cognitive assessment, we may not find strong ideal point response processes as individuals are not motivated to be accurate. Therefore, the kind and level of motivation could be moderators for the response process.

#### Meaning of Construct Continuum

A difference between cognitive and non-cognitive constructs is that the construct continuum reflects different underlying meanings. For cognitive constructs, the continuum reflects varying levels of *difficulty* in the sense that the nature of these constructs usually requires an individual to harness resources to produce a correct response and demonstrate maximal performance. This is reflected in the use of the term “item *difficulty* parameters” in IRT models when assessing constructs requiring performance (Schatschneider et al., 1999; Embretson and Gorin, 2001; Maller, 2001; Gorin, 2005; Daniel and Embretson, 2010). For non-cognitive constructs, the construct continuum is better understood as various *locations*, since the “correct” response is simply that which most accurately reflects one’s position along the latent trait. This is reflected in the references to “item *location* parameters” when IRT is applied to non-cognitive constructs (Reise et al., 2001; Reise and Haviland, 2005; Vittersø et al., 2005; Gomez, 2008; Weekers and Meijer, 2008; Zampetakis, 2010; Reise et al., 2011).

#### Polarity

Another feature that distinguishes the construct continuum for cognitive constructs is the construct poles (Tay and Drasgow, 2012). Cognitive construct poles are unipolar, ranging from low to high and implying absolute absence to presence of a latent trait, respectively. Unipolarity is also reflected in how cognitive ability assessment indicators are not reverse scored. On the other hand, non-cognitive construct poles can be unipolar or bipolar. Bipolar constructs—or constructs that are assessed in a bipolar manner—construe the construct poles as assessing a construct on one end and its opposite on the other and are seen in multiple areas of non-cognitive construct assessment, including personality (e.g., “fearful, apprehensive” to “relaxed, unconcerned, cool” [Neuroticism-anxiousness]; Samuel and Widiger, 2004; Mullins-Sweatt et al., 2006), attitudes (e.g., “bad” to “good”; Fishbein and Raven, 1962), and affect (e.g., negative affect to positive affect; Russell and Carroll, 1999). This particular issue is important since ideal point models were primarily developed in the context of bipolar attitudinal research (Thurstone, 1928). Within this context, ideal point models arguably provide a straightforward way to scale items and individuals. In contrast, it is difficult to use dominance models to scale individuals along the length of the bipolar continuum because Likert-type scaling and reverse scoring of items lead to the removal of items located near the middle of the continuum. By extension, it is likely that individuals will demonstrate more of an ideal point response process with bipolar constructs that have items along the continuum as shown in past research (Tay et al., 2009; Tay and Kuykendall, 2016).

#### Item Response Process and Models

Given the aforementioned issues/factors, some have proposed that the item response processes underlying cognitive and non-cognitive constructs reflect a hurdle algorithm and a matching algorithm, respectively (Tay and Drasgow, 2012). These algorithms are general descriptive processes of how individuals engage with scale items. The hurdle algorithm of response process likens assessment items to hurdles to overcome, which is common in cognitive ability assessments, resulting in the more appropriate use of dominance response models. The matching algorithm has been proposed for non-cognitive constructs and describes how individuals seek to implicitly or explicitly decide on whether the presented statement matches their trait level as modeled with ideal point response models. For example, an individual will decide on whether the statement “happy” fully matches their affective state—if they are too above (e.g., “elated”) or below (e.g., “content”), there is not a full match and they will not have the highest probability of responding positively to the statement.

It is critical to note that these algorithms are fundamentally posited at the within-person level (see LaPalme et al., 2018). We further posit that each and every individual engages in a similar hurdle or matching algorithm when responding to assessment items. Gathering within-person level data (e.g., pairwise comparisons or repeated assessments) would reveal individual response curves that have dominance or ideal point shapes. However, the current measurement models often used to infer these descriptive processes have generally used between-person level data. These are not necessarily commensurate and it is an empirical gap that needs to be addressed. The development of within-person models can more fully allow researchers to explore individual-level moderators of item response processes. For example, there is research suggesting that there are individual differences in how selective people are using words to describe emotions (Barrett et al., 2001). These differences in selectivity may affect the extent to which we observe ideal point responding.

Having reviewed the theoretical differences between cognitive and non-cognitive constructs, we now turn to a discussion of factors that affect whether respondents engage in the ideal point response process.

### Factors That Influence Whether Respondents Engage in the Ideal Point Response Process

When respondents introduce systemic variance into their responses, they distort what would otherwise be an accurate portrayal of themselves. This is generally referred to as response effects (Schwarz, 2007) or response bias (Paulhus, 1991). We make a distinction between two general types of response effects and focus our discussion on two factors (i.e., ability and motivation) that shape the degree to which respondents engage in the ideal point response process when responding non-cognitive constructs items.

#### Amotivated Response Effects

Within the literature on cognitive aspects of survey methodology (Jabine et al., 1984), Krosnick (1991, 1999) proposed that respondents fall on a continuum of optimizing versus satisficing *response strategy* that describes the accuracy and thoroughness with which they engage in the four steps of cognitive processes (i.e., question comprehension, memory retrieval, judgment integration, and response selection; Sudman et al., 1996; Tourangeau et al., 2000) when formulating responses to items. The degree of satisficing respondents engaged in can parsimoniously explain the vast and varied empirical support for “amotivated” response effects: response order effects (e.g., primacy effects for first option on rating scale within range of preference), acquiescence effects (i.e., tendency to agree or endorse), nondifferentiation (straightlining or giving the same response across items), and part of the explanation for understanding the nature of “no opinion” options (Krosnick, 1999). Three factors, each with their own multiple contributing factors, influence the degree of optimization: task difficulty (e.g., affected by ability to interpret item), respondent ability (e.g., function of aptitude in doing complex mental tasks), and respondent motivation (e.g., including personal importance of the topic the item taps) (Krosnick, 1991).

Thus, the more that a respondent engages in optimization rather than satisficing, the more we would expect their responses to reflect an ideal point response process when answering non-cognitive construct items. Indeed, a recent investigation of within-person response processes when responding to non-cognitive construct (i.e., attitude, affect, and personality) items showed that a) responses tended to consistently conform more to an ideal point model than a dominance model and b) those high in verbal ability or conscientiousness were more likely to engage in an ideal point response process (LaPalme et al., 2018). As it relates to the factors leading to optimization in responding, verbal ability would represent respondent ability and also affect task difficulty, and conscientiousness would reflect motivation to perform well (i.e., thoroughly and accurately) on the task. These individual differences increase ideal point response process, perhaps because they aid in optimization.

#### Motivated Response Effects

Social desirability bias or responding (Paulhus, 1991; Krosnick, 1999) is the tendency to give responses that reflect favorably on the respondent, whether consciously or unconsciously motivated (Paulhus and John, 1998). Models of faking behavior in this area generally propose that faking is determined by ability and motivation to fake (Snell et al., 1999; McFarland and Ryan, 2000, 2006). Regarding ability to fake, knowledge about the nature of the construct being assessed (e.g., Cunningham et al., 1994; Mcfarland and Ryan, 2000), familiarity with what constitutes the criteria for a “good” response (e.g., job characteristics for which a personality test is being used as a selection tool; Furnham, 1990; Mahar et al., 1995; Martin et al., 2002), and perhaps general or emotional intelligence (Snell et al., 1999), as well as others (e.g., opportunity to fake, item transparency; McFarland and Ryan, 2006) affect ability to fake. In terms of motivation to fake, things that theoretically or empirically increase motivation to fake include contextual factors such as administration in a high-stakes selection context; respondents’ attitudes about faking; perceived subjective norms surrounding faking, including perceptions of others’ behaviors and attitudes about faking, their willingness to fake, how competitive they are for a position, and of organizational fairness; expectancy regarding success; and importance of assessment outcome (Snell et al., 1999; Mcfarland and Ryan, 2000; McFarland and Ryan, 2006; Goffin and Boyd, 2009).

To the extent that these contextual and individual factors are present, we would expect to find greater prevalence of dominance response processes as compared to ideal point response processes. This is because individuals may view these self-reported typical behavior items as hurdles to overcome. As such, in high-stakes testing situations, past research has found qualified support of different response processes to personality data based on differential item functioning analysis (O’Brien and LaHuis, 2011). This is not to say that ideal point models cannot be applied under such conditions as researchers have applied ideal point models to assess faking, as evidenced in shifts in the item location parameters (Scherbaum et al., 2013).

### Review of Practical Implications

The theoretical framework in the previous section provides a way for researchers to identify the key factors/issues associated with the ideal point measurement of non-cognitive constructs. Research has shown that ideal point measurement models fit well to the non-cognitive constructs of attitudes (Roberts et al., 1999), personality (Stark et al., 2006), vocational interests (Tay et al., 2009), and affect (Tay and Kuykendall, 2016). Yet, are ideal point measurement models truly needed apart from increasing measurement fidelity given the longstanding use of dominance measurement models for non-cognitive constructs? What are the practical implications of using ideal point models?

Connecting basic research with practices in applied research can be illustrative. Earlier research investigated whether rank ordering of personality scores changes with dominance and ideal point methods (Stark et al., 2006). Both methods produced very similar scores as evidenced by the high correlations across different personality scales in the overall sample (*r’s ≈* 0.98). Stark et al. (2006) suggested that one possible reason for this strong association is that current personality scales have been developed using dominance methods. Dominance methods for scale development, including the use of scale development statistics (e.g., item-total correlations), exclude middling items, which conform less well to ideal point response model assumptions. As a result, scoring methods may not yield very different scores.

However, the ideal point method scoring has serious practical implications in applied work, such as in employee selection in the work context. Further comparisons of the two scoring methods by Stark et al. (2006) revealed that while the overall sample may not have dramatically different scores, for some personality traits, the scores may dramatically differ when examining the top 100 ideal point scorers (out of 2,000), which is a typical selection scenario based on cutoff scores in personnel selection settings. In this subset, the dominance scores were actually negatively correlated (*r’s ≈* −0.40) with the ideal point scores. This suggests that there may not be correspondence across the entire continuum between dominance versus ideal point scoring methods, particularly at the higher levels.

Indeed, Carter et al. (2014; Study 3) used data collected from a consulting firm to compare ideal point-based scoring against a few dominance-based scoring methods. They used all scoring methods to a) calculate conscientiousness scores and used those scores to b) predict values in several job performance metrics using coefficients obtained from a prior study, c) ranked and selected the top 100 candidates on the basis of those predicted job performance metrics, and finally d) examined the incidence rates of different types of actual turnover for the individuals selected across the different job performance metrics as predicted from conscientiousness scores calculated from ideal point- versus dominance-based methods. They found that ideal point-based scoring models generally decreased the selection of individuals who turned over for reasons that would be harmful to the organization (e.g., person-environment fit, performance and behavioral issues, and job abandonment).

The practical implications of using ideal point models extend well beyond the work context. For example, ideal point models have been applied to the medical context to create a scale to measure the degree of control patients want to exercise in making treatment decisions when facing a serious illness (Control Preferences Scale; Degner et al., 1997). Given that a patient likely has less knowledge about treatment options for their disease than their doctor, the patient may want to defer to them to some degree in treatment decisions. This consideration and its conceptualization as a preference implicate an ideal point model for this non-cognitive construct, and the resultant measure based on that model has applications to both theory and practice (Degner et al., 1997) as a tool for measurement and clinical assessment. A measure that more accurately models patients’ desire for control in healthcare decision-making has important consequences for both their health outcomes and their sense of agency.

Another critical area is the assessment of construct dimensionality. As mentioned earlier, past research has shown that misapplying dominance models (e.g., principal components analysis or factor analysis) to unidimensional bipolar scales leads to an additional spurious orthogonal factor (Davison, 1977). This may be a significant methodological oversight that has led to psychological phenomena traditionally thought to be bipolar such as attitudes and affect being reconceptualized as bivariate (Cacioppo et al., 1997). Indeed, more recent research suggests that ideal point models more accurately model unidimensional bipolarity as in the case of affect (Tay and Drasgow, 2012; Tay and Kuykendall, 2017). This is not to say that ideal point modeling is the sole reason or necessarily the most pervasive reason; but it is a significant factor that should be considered in construct dimensionality and construct validation work, especially for self-reported typical behaviors including attitudes, emotions, interests, and the like.

### Future Directions

There are several future directions for research on non-cognitive assessments with respect to ideal point models. One area of research is the continued development of ideal point measurement models. This would include developing generalized multidimensional ideal point measurement models for different response scales. Work has begun in this area for binary data (Maydeu-Olivares et al., 2006). Apart from developing these models, we also need to concomitantly develop ways (e.g., fit indices) to determine how well they fit the data (e.g., Tay et al., 2011). Many fit indices in structural equation modeling and IRT have been developed based on dominance response models, which may not be as appropriate for non-cognitive constructs. Having robust models and methods will serve to encourage new scale development techniques and ways to evaluate scales (e.g., measurement equivalence; Wang et al., 2013) that are based on ideal point response assumptions.

Another area of research is scale creation—or the development of scale creation tools and procedures based on ideal point models and principles (Chernyshenko et al., 2007). Developments have been made to assess the unidimensionality of an ideal point continuum (Habing et al., 2005), but more work will be needed to develop multidimensional assessments as an analog to factor analysis frequently used in scale creation and validation. Additionally, conventional reliability estimates rely on dominance assumptions that will require new forms accounting for ideal point model assumptions. The creation of ideal point scales and estimation of scale item parameters will also be instructive for setting priors in Bayesian estimation of ideal point models that are becoming more popular.

We also believe that the issue of assessing the bipolarity of non-cognitive constructs will become more important over time due to several factors. The rise of positive psychology and the introduction or emphasis of positive constructs (Seligman and Csikszentmihalyi, 2000) has led to questions as to whether these are novel constructs or extensions of the old (Seligman and Csikszentmihalyi, 2001; Held, 2005; Kashdan et al., 2008; Kristjánsson, 2010; Noftle et al., 2011; Wong, 2011). In accordance with the rise of positive psychology, researchers are also increasingly interested in the differences and interplay between “bright” and “dark” traits (Judge et al., 2009; Resick et al., 2009; Furnham et al., 2012; Furnham and Crump, 2014; Harms and Spain, 2015). Yet, there are similar questions as to whether these traits should be understood as parts of a common continuum or orthogonal dimensions. Psychology is also moving toward a greater emphasis on consolidating and replicating findings (Schmidt, 2009; Schooler, 2014; Maxwell et al., 2015), and we need to use methodologies appropriate for addressing issues of non-cognitive construct continua.

Continued work will also need to demonstrate when and whether there are practical consequences to applying dominance models to non-cognitive constructs. Based on our theoretical framework, we posit that there may be conditions in which there may be less ideal point responding, such as in high-stakes testing contexts. This would lead to less accurate self-descriptions of typical behavior. Examining the contextual factors that contribute to which item response process (i.e., hurdle vs. matching algorithm) is chosen is an important future line of research. Future research should also investigate if all non-cognitive constructs similarly evoke the hypothesized item response process. Implicit-type construct or those that tend to lead to biased responses, such as socially desirable constructs, may not lead to ideal point responding.

Ideal point modeling and measurement does not exist in a vacuum devoid of conceptual considerations. Rather it occurs in the broader context of understanding our constructs and the construct continua to which individuals are responding to. For example, individuals may not agree that they merely had a “good” day because they had a “terrific” day or conversely they had an “average” day. Accurately applying ideal point models and developing ideal point models require a deeper understanding of the full span of a construct continuum, what its poles are (at both the high and low end) and what is the nature of the continuum (e.g., frequency, intensity, etc.). More recent conceptual work has proposed the concept of *continuum specification* in construct validity and validation where researchers will need to better define and operationalize constructs explicitly with regard to their continua (Samuel and Tay, 2018; Tay and Jebb, 2018).

## Conclusion

The goal of our paper has been to promote the use of ideal point models for non-cognitive assessments or measures of self-reported typical behaviors. To this end, we have provided a historical and theoretical backdrop for ideal point response processes and models. We then provided an integrative framework for understanding why ideal point models are most appropriate for non-cognitive constructs. Finally, we reviewed the potential practical consequences of not accounting for the ideal point response processes in our measurement models. It is our hope that it has become clear that ideal point models may be more ideal for non-cognitive construct assessment than dominance models and that this recognition spurs continued advancements in this cutting edge area of psychometric research.

## Author Contributions

LT contributed to the conception and design of the manuscript. LT wrote the first draft of the manuscript; VN contributed to subsequent drafts and added sections. VN and LT contributed to manuscript revision, read, and approved submitted version.

### Conflict of Interest Statement

The authors declare that the research was conducted in the absence of any commercial or financial relationships that could be construed as a potential conflict of interest.

## References

[ref1] Andrich D. Luo G. (1993). A hyperbolic cosine latent trait model for unfolding dichotomous single-stimulus responses. Appl. Psychol. Meas. 17, 253–276.

[ref2] Barrett L. F. Gross J. Christensen T. C. Benvenuto M. (2001). Knowing what you’re feeling and knowing what to do about it: mapping the relation between emotion differentiation and emotion regulation. Cognit. Emot. 15, 713–724. 10.1080/02699930143000239

[ref3] Bock R. D. (1997). A brief history of item response theory. Educational measurement: issues and practice. 16, 21–33. 10.1016/S0962-8924(97)01160-4, PMID: 17709014

[ref4] Borsboom D. Mellenbergh G. J. van Heerden J. (2003). The theoretical status of latent variables. Psychol. Rev. 110, 203–219. 10.1037/0033-295X.110.2.203, PMID: 12747522

[ref5] Borsboom D. Mellenbergh G. J. van Heerden J. (2004). The concept of validity. Psychol. Rev. 111, 1061–1071. 10.1037/0033-295X.111.4.1061, PMID: 15482073

[ref6] Bradburn N. M. (1969). The structure of psychological well-being. Chicago: Aldine.

[ref7] Brayfield A. Rothe H. (1951). An index of job satisfaction. J. Appl. Psychol. 35, 307–311. 10.1037/h0055617. Retrieved from: http://psycnet.apa.org/journals/apl/35/5/307/

[ref8] Cacioppo J. T. Bernston G. G. (1994). Relationship between attitudes and evaluative space: a critical review, with emphasis on the separability of positive and negative substrates. Psychol. Bull. 115, 401–423. 10.1037/0033-2909.115.3.401

[ref9] Cacioppo J. T. Gardner W. L. Bernston G. G. (1997). Beyond bipolar conceptualizations and measures: the case of attitudes and evaluative space. Personal. Soc. Psychol. Rev. 1, 3–25. 10.1207/s15327957pspr0101_2 15647126

[ref10] Cao M. Song Q. C. Tay L. (2018). Detecting curvilinear relationships: a comparison of scoring approaches based on different item response models. Int. J. Test. 18, 178–205. 10.1080/15305058.2017.1345913

[ref11] Carter N. T. Dalal D. K. Boyce A. S. O’Connell M. S. Kung M.-C. Delgado K. M. (2014). Uncovering curvilinear relationships between conscientiousness and job performance: how theoretically appropriate measurement makes an empirical difference. J. Appl. Psychol. 99, 564–586. 10.1037/a0034688, PMID: 24188394

[ref12] Carter N. T. Dalal D. K. Guan L. Lopilato A. C. Withrow S. A. Carter N. T. (2017). Item response theory scoring and the detection of curvilinear relationships. Psychol. Methods. 22, 191–203. 10.1037/met0000101, PMID: 27819433

[ref13] Carter N. T. Guan L. Maples J. L. Williamson R. L. Miller J. D. (2016). The downsides of extreme conscientiousness for psychological well-being: the role of obsessive compulsive tendencies. J. Pers. 84, 510–522. 10.1111/jopy.12177, PMID: 25858019

[ref14] Chernyshenko O. S. Stark S. Drasgow F. Roberts B. W. (2007). Constructing personality scales under the assumptions of an ideal point response process: toward increasing the flexibility of personality measures. Psychol. Assess. 19, 88–106. 10.1037/1040-3590.19.1.88, PMID: 17371125

[ref15] Coombs H. C. (1964). A theory of data. New York: John Wiley. 10.1038/203523a0, PMID:

[ref16] Cronbach L. J. Meehl P. E. (1955). Construct validity in psychological tests. Psychol. Bull. 52, 281–302. 10.1037/h0040957, PMID: 13245896

[ref17] Cunningham M. R. Wong D. T. Barbee A. P. (1994). Self-presentation dynamics on overt integrity tests: experimental studies of the Reid Report. J. Appl. Psychol. 79, 643–658. 10.1037/0021-9010.79.5.643, PMID: 7989274

[ref18] Daniel R. C. Embretson S. E. (2010). Designing cognitive complexity in mathematical problem-solving items. Appl. Psychol. Meas. 34, 348–364. 10.1177/0146621609349801

[ref19] Davison M. L. (1977). On a metric, unidimensional unfolding model for attitudinal and developmental data. Psychometrika. 42, 523–548. 10.1007/BF02295977

[ref20] Degner L. F. Sloan J. A. Venkatesh P. (1997). The control preferences scale. Can. J. Nurs. Res. 29, 21–43, PMID: . 9505581

[ref21] Drasgow F. Chernyshenko O. S. Stark S. (2010). 75 years after Likert: Thurstone was right! Industrial and organizational psychology*:* Perspect. Sci. Practice. 3, 465–476.

[ref22] Edwards J. R. Bagozzi R. P. (2000). On the nature and direction of relationships between constructs and measures. Psychol. Methods, 5, 155–174. 10.1037/1082-989X.5.2.155, PMID: 10937327

[ref23] Embretson S. Gorin J. (2001). Improving construct validity with cognitive psychology principles. J. Educ. Meas. 38, 343–368. 10.1111/j.1745-3984.2001.tb01131.x

[ref24] Fishbein M. Raven B. H. (1962). The AB scales: an operational definition of belief and attitude. Hum. Relat. 15, 35–44.

[ref25] Furnham A. (1990). Faking personality questionnaires: fabricating different profiles for different purposes. Curr. Psychol. Res. Rev. 9, 46–55. 10.1007/BF02686767

[ref26] Furnham A. Crump J. (2014). A bright side, facet analysis of schizotypal personality disorder: the relationship between the HDS imaginative factor, the NEO-PI-R personality trait facets in a large adult sample. Think. Skills Creat. 11, 42–47. 10.1016/j.tsc.2013.10.001

[ref27] Furnham A. Trickey G. Hyde G. (2012). Bright aspects to dark side traits: dark side traits associated with work success. Personal. Individ. Differ. 52, 908–913. 10.1016/j.paid.2012.01.025

[ref28] Goffin R. D. Boyd A. C. (2009). Faking and personality assessment in personnel selection: advancing models of faking. Can. Psychol. 50, 151–160. 10.1037/a0015946

[ref29] Gomez R. (2008). Parent ratings of the ADHD items of the disruptive behavior rating scale: analyses of their IRT properties based on the generalized partial credit model. Personal. Individ. Differ. 45, 181–186. 10.1016/j.paid.2008.04.001

[ref30] Gorin J. S. (2005). Manipulating processing difficulty of reading comprehension questions: the feasibility of verbal item generation. J. Educ. Meas. 42, 351–373. 10.1111/j.1745-3984.2005.00020.x

[ref31] Grant A. M. Schwartz B. (2011). Too much of a good thing: the challenge and opportunity of the inverted-U. Perspect. Psychol. Sci. 6, 61–76. 10.1177/1745691610393523, PMID: 26162116

[ref32] Greenwald A. G. (2012). There is nothing so theoretical as a good method. Perspect. Psychol. Sci. 7, 99–108. 10.1177/1745691611434210, PMID: 26168438

[ref33] Greenwald A. G. McGhee D. E. Schwartz J. L. K. (1998). Measuring individual differences in implicit cognition: the implicit association test. J. Pers. Soc. Psychol. 74, 1464–1480. 10.1037/0022-3514.74.6.1464, PMID: 9654756

[ref34] Habing B. Finch H. Roberts J. (2005). A Q3 statistic for unfolding item response theory models: assessment of unidimensionality with two factors and simple structure. Appl. Psychol. Meas. 29, 457–471. 10.1177/0146621604279550

[ref35] Harms P. D. Spain S. M. (2015). Beyond the bright side: dark personality at work. Appl. Psychol. Int. Rev. 64, 15–24. 10.1111/apps.12042

[ref36] Hegarty M. Richardson A. E. Montello D. R. Lovelace K. Subbiah I. (2002). Development of a self-report measure of environmental spatial ability. Intelligence. 30, 425–447. 10.1016/S0160-2896(02)00116-2

[ref37] Held B. S. (2005). The “virtues” of positive psychology. J. Theor. Philos. Psychol. 25, 1–34. 10.1037/h0091249

[ref38] Jabine T. Straf M. Tanur J. Tourangeau R. (1984). Cognitive aspects of survey methodology: building a bridge between disciplines. in Advanced research seminar on cognitive aspects of survey methodology. (Saint Michaels and Baltimore: National Academy Press).

[ref39] James L. R. LeBreton J. M. (2012). Assessing the implicit personality through conditional reasoning. Washington, DC: American Psychological Association. 10.1177/2045125312453158, PMID:

[ref40] Judge T. A. Piccolo R. F. Kosalka T. (2009). The bright and dark sides of leader traits: a review and theoretical extension of the leader trait paradigm. Leadersh. Q. 20, 855–875. 10.1016/j.leaqua.2009.09.004

[ref41] Kashdan T. B. Biswas-Diener R. King L. A. (2008). Reconsidering happiness: the costs of distinguishing between hedonics and eudaimonia. J. Posit. Psychol. 3, 219–233. 10.1080/17439760802303044

[ref42] Kristjánsson K (2010). Positive psychology, happiness, and virtue: the troublesome conceptual issues. Rev. Gen. Psychol. 14, 296–310. 10.1037/a0020781

[ref43] Krosnick J. A. (1991). Response strategies for coping with the cognitive demands of attitute measure in surveys. Appl. Cogn. Psychol. 5, 213–236. 10.1002/acp.2350050305

[ref44] Krosnick J. A. (1999). Survey research. Annu. Rev. Psychol. 50, 537–567. 10.4135/9781849209984, PMID: 15012463

[ref45] LaPalme M. Tay L. Wang W. (2018). A within-person examination of the ideal-point response process. Psychol. Assess. 30, 567–581. 10.1037/pas0000499, PMID: 28557481

[ref46] Larsen J. T. Norris C. J. McGraw A. P. Hawkley L. C. Cacioppo J. T. (2009). The evaluative space grid: a single-item measure of positivity and negativity. Cognit. Emot. 23, 453–480. 10.1080/02699930801994054

[ref47] Likert R. (1932). A technique for the measurement of attitudes. Arch. Psychol. 140, 5–53.

[ref48] Lovell C (1945). A study of the factor structure of thirteen personality variables. Educ. Psychol. Meas. 5, 335–350.

[ref49] Mahar D. Cologon J. Duck J. (1995). Response strategies when faking personality questionnaires in a vocational selection setting. Personal. Individ. Differ. 18, 605–609. 10.1016/0191-8869(94)00200-C

[ref50] Maller S. J. (2001). Differential item functioning in the WISC-III: item parameters for boys and girls in the national standardization sample. Educ. Psychol. Meas. 61, 793–817. 10.1177/00131640121971527

[ref51] Martin B. A. Bowen C.-C. Hunt S. T. (2002). How effective are people at faking on personality questionnaires ? Personal. Individ. Differ. 32, 247–256. 10.1016/S0191-8869(01)00021-6

[ref52] Maxwell S. E. Lau M. Y. Howard G. S. (2015). Is psychology suffering from a replication crisis? Am. Psychol. 70, 487–498. 10.1037/a0039400, PMID: 26348332

[ref53] Maydeu-Olivares A. Hernández A. McDonald R. P. (2006). A multidimensional ideal point item response theory model for binary data. Multivar. Behav. Res. 41, 445–471. 10.1207/s15327906mbr4104_2, PMID: 26794914

[ref54] Mcfarland L. A. Ryan A. M. (2000). Variance in faking across noncognitive measures. J. Appl. Psychol. 85, 812–821. 10.1037/0021-9010.85.5.812, PMID: 11055152

[ref55] McFarland L. A. Ryan A. M. (2006). Toward an integrated model of applicant faking behavior. J. Appl. Soc. Psychol. 36, 979–1016. 10.1111/j.0021-9029.2006.00052.x

[ref56] Mullins-Sweatt S. N. Jamerson J. E. Samuel D. B. Olson D. R. Widiger T. A. (2006). Psychometric properties of an abbreviated instrument of the five-factor model. Assessment. 13, 119–137. 10.1177/1073191106286748, PMID: 16672728

[ref57] Noftle E. E. Schnitker S. A. Robins R. W. (2011). Character and personality: connections between positive psychology and personality psychology. in Designing positive psychology: taking stock and moving forward. eds. Sheldon K. M. Kashdan T. B. Steger M. F. (New York, NY: Oxford University Press), 207–227.

[ref58] O’Brien E. LaHuis D. M. (2011). Do applicants and incumbents respond to personality items similarly? A comparison of dominance and ideal point response models. Int. J. Sel. Assess. 19, 109–118. 10.1111/j.1468-2389.2011.00539.x

[ref59] Paulhus D. L. (1991). Measurement and control of response bias. in Measures of personality and social psychological attitudes 1 eds. Robinson J. P. Shaver P. R. Wrightsman L. S. (San Diego, CA: Academic Press), 17–59. Retrieved from: http://doi.apa.org/psycinfo/1991-97206-001

[ref60] Paulhus D. L. John O. P. (1998). Egoistic and moralistic biases in self-perception: the interplay of self-deceptive styles with basic traits and motives. J. Pers. 66, 1025–1060. 10.1111/1467-6494.00041

[ref61] Pierce J. R. Aguinis H. (2011). The Too-Much-of-a-Good-Thing Effect in management. J. Manag. 39, 313–338. 10.1177/0149206311410060

[ref62] Reise S. P. Haviland M. G. (2005). Item response theory and measurement of clinical change. J. Pers. Assess. 84, 228–238. 10.1207/s15327752jpa8403, PMID: 15907159

[ref63] Reise S. P. Horan W. P. Blanchard J. J. (2011). The challenges of fitting an item response theory model to the social anhedonia scale. J. Pers. Assess. 93, 213–224. 10.1080/00223891.2011.558868, PMID: 21516580 PMC3117299

[ref64] Reise S. P. Smith L. Furr M. R. (2001). Invariance on the NEO PI-R neuroticism scale. Multivar. Behav. Res. 36, 83–110. 10.1207/S15327906MBR3601

[ref65] Resick C. J. Whitman D. S. Weingarden S. M. Hiller N. J. (2009). The bright-side and the dark-side of CEO personality: examining core self-evaluations, narcissism, transformational leadership, and strategic influence. J. Appl. Psychol. 94, 1365–1381. 10.1037/a0016238, PMID: 19916649

[ref66] Rest J. R. (1986). DIT: manual for the defining issues test. Center for the Study of Ethical Development: University of Minnesota. 10.1016/0014-4827(86)90212-0, PMID:

[ref67] Roberts J. S. (2001). GGUM2000: estimation of parameters in the generalized graded unfolding model. Appl. Psychol. Meas. 25, 38.

[ref68] Roberts J. S. (2008). Modified likelihood-based item fit statistics for the Generalized Graded Unfolding Model. Appl. Psychol. Meas. 32, 407–423. 10.1177/0146621607301278

[ref69] Roberts J. S. Donoghue J. R. Laughlin J. E. (2000). A general item response theory model for unfolding unidimensional polytomous responses. Appl. Psychol. Meas. 24, 3–32. 10.1177/01466216000241001

[ref70] Roberts J. S. Donoghue J. R. Laughlin J. E. (2002). Characteristics of MML/EAP parameter estimates in the generalized graded unfolding model. Appl. Psychol. Meas. 26, 192–207. 10.1177/01421602026002006

[ref71] Roberts J. S. Fang H. Cui W. Wang Y. (2004). GGUM2004: a windows based program to estimate parameters in the generalized graded unfolding model. Appl. Psychol. Meas. 30, 64–65. 10.1177/0146621605280141

[ref72] Roberts J. S. Laughlin J. E. Wedell D. H. (1999). Validity issues in the Likert and Thurstone approaches to attitude measurement. Educ. Psychol. Meas. 59, 211–233. 10.1177/00131649921969811

[ref73] Russell J. A. (1979). Affective space is bipolar. J. Pers. Soc. Psychol. 37, 345–356. 10.1037/0022-3514.37.3.345

[ref74] Russell J. A. Carroll J. M. (1999). On the bipolarity of positive and negative affect. Psychol. Bull. 125, 3–30. 10.1037/0033-2909.125.1.3, PMID: 9990843

[ref75] Sackett P. R. Zedeck S. Fogli L. (1988). Relations between measures of typical and maximum job performance. J. Appl. Psychol. 73, 482–486. 10.1037/0021-9010.73.3.482

[ref76] Samuel D. B. Tay L. (2018). Aristotle’s golden mean and the importance of bipolarity for personality models: a commentary on “Personality traits and maladaptivity: unipolarity versus bipolarity”. J. Pers. 10.1111/jopy.12383, PMID: 29577297

[ref77] Samuel D. B. Widiger T. A. (2004). Clinicians’ personality descriptions of prototypic personality disorders. J. Personal. Disord. 18, 286–308. 10.1521/pedi.18.3.286.35446, PMID: 15237048

[ref78] Schatschneider C. Francis D. J. Foorman B. R. Fletcher J. M. Mehta P. (1999). The dimensionality of phonological awareness: an application of item response theory. J. Educ. Psychol. 91, 439–449. 10.1037/0022-0663.91.3.439

[ref79] Scherbaum C. A. Sabet J. Kern M. J. Agnello P. (2013). Examining faking on personality inventories using unfolding item response theory models. J. Pers. Assess. 95, 207–216. 10.1080/00223891.2012.725439, PMID: 23030769

[ref80] Schmidt S (2009). Shall we really do it again? The powerful concept of replication is neglected in the social sciences. Rev. Gen. Psychol. 13, 90–100. 10.1037/a0015108

[ref81] Schooler J. (2014). Metascience could rescue the replication crisis. Nature 515:9. 10.1038/515009a, PMID: 25373639

[ref82] Schreiber R. J. Smith R. G. Harrell T. W. (1952). A factor analysis of employee attitudes. J. Appl. Psychol. 36, 247–250. 10.1037/h0055597

[ref83] Schwarz N. (2007). Cognitive aspects of survey methodology. Appl. Cogn. Psychol. 21, 1057–1075. 10.1002/acp0

[ref84] Seligman M. E. P. Csikszentmihalyi M. (2000). Positive psychology: an introduction. Am. Psychol. 55, 5–14. 10.1037/0003-066X.55.1.5, PMID: 11392865

[ref85] Seligman M. E. P. Csikszentmihalyi M. (2001). Reply to comments (editorial). Am. Psychol. 56, 89–90. 10.1037//0003-066X.56.1.89

[ref86] Snell A. F. Sydell E. J. Lueke S. B. (1999). Towards a theory of applicant faking: integrating studies of deception. Hum. Resour. Manag. Rev. 9, 219–242. 10.1016/S1053-4822(99)00019-4

[ref87] Spearman C. (1904). General intelligence, objectively determined and measured. Am. J. Psychol. 15, 201–293. 10.2307/1412107

[ref88] Stark S. Chernyshenko O. S. Drasgow F. Williams B. A. (2006). Examining assumptions about item responding in personality assessment: should ideal point methods be considered for scale development and scoring? J. Appl. Psychol. 91, 25–39. 10.1037/0021-9010.91.1.25, PMID: 16435936

[ref89] Sudman S. Bradburn N. M. Schwarz N. (1996). Thinking about answers: the application of cognitive processes to survey methodology. San Francisco: Jossey-Bass.

[ref90] Tay L. Ali U. S. Drasgow F. Williams B. A. (2011). Fitting IRT models to dichotomous and polytomous data: assessing the relative model–data fit of ideal point and dominance models. Appl. Psychol. Meas. 35, 280–295. 10.1177/0146621610390674

[ref91] Tay L. Drasgow F. (2012). Theoretical, statistical, and substantive issues in the assessment of construct dimensionality: accounting for the item response process. Organ. Res. Methods, 15, 363–384. 10.1177/1094428112439709

[ref92] Tay L. Drasgow F. Rounds J. Williams B. (2009). Fitting measurement models to vocational interest data: are dominance models ideal? J. Appl. Psychol. 94, 1287–1304. 10.1037/a0015899, PMID: 19702371

[ref93] Tay L. Jebb A. T. (2018). Establishing construct continua in construct validation: the process of continuum specification. Adv. Methods Pract. Psychol. Sci. 1, 375–388. 10.1177/2515245918775707

[ref94] Tay L. Kuykendall L. (2017). Why self-reports of happiness and sadness may not necessarily contradict bipolarity: a psychometric review and proposal. Emot. Rev. 9, 146–154. 10.1177/1754073916637656

[ref95] Thurstone L. L. (1925). A method of scaling psychological and educational tests. J. Educ. Psychol. 16, 433–451. 10.1037/h0073357

[ref96] Thurstone L. L. (1927a). A law of comparative judgment. Psychol. Rev. 34, 273–286.

[ref97] Thurstone L. L. (1927b). Psychophysical analysis. Am. J. Psychol. 38, 368–389. 3322058

[ref98] Thurstone L. L. (1928). Attitudes can be measured. Am. J. Psychol. 33, 529–554.

[ref99] Tourangeau R. Rips L. C. Rasinski K. (2000). The psychology of survey response. Cambridge: Cambridge University Press.

[ref100] Tyler F. T. (1951). A factorial analysis of fifteen MMPI scales. J. Consult. Psychol. 15, 451–456. 10.1037/h0054518, PMID: 14888748

[ref101] Vittersø J. Biswas-Diener R. Diener E. (2005). The divergent meanings of life satisfaction: item response modeling of the satisfaction with life scale in Greenland and Norway. Soc. Indic. Res. 74, 327–348. 10.1007/s11205-004-4644-7

[ref102] Wang W. Tay L. Drasgow F. (2013). Detecting differential item functioning of polytomous items for an ideal point response process. Appl. Psychol. Meas. 37, 316–335. 10.1177/0146621613476156

[ref103] Watson D. Tellegen A. (1985). Toward a consensual structure of mood. Psychol. Bull. 98, 219–235. 10.1037/0033-2909.98.2.219, PMID: 3901060

[ref104] Weekers A. M. Meijer R. R. (2008). Scaling response processes on personality items using unfolding and dominance models: an illustration with a Dutch dominance and unfolding personality inventory. Eur. J. Psychol. Assess. 24, 65–77. 10.1027/1015-5759.24.1.65

[ref105] Wiese C. W. Tay L. Duckworth A. L. D’Mello S. Kuykendall L. Hofmann W., (2018). Too much of a good thing? Exploring the inverted-U relationship between self-control and happiness. J. Pers. 86, 380–396. 10.1111/jopy.12322, PMID: 28480971 PMC5677575

[ref106] Wong P. T. P. (2011). Positive psychology 2.0: towards a balanced interactive model of the good life. Can. Psychol. 52, 69–81. 10.1037/a0022511

[ref107] Zampetakis L. A. (2010). Unfolding the measurement of the creative personality. J. Creat. Behav. 44, 105–123. 10.1002/j.2162-6057.2010.tb01328.x

[ref108] Zinnes J. L. Griggs R. A. (1974). Probabilistic, multidimensional unfolding analysis. Psychometrika, 39, 327–350. 10.1007/BF02291707

